# Safety and satisfaction of myopic small-incision lenticule extraction combined with monovision

**DOI:** 10.1186/s12886-018-0794-2

**Published:** 2018-05-31

**Authors:** Dan Fu, Li Zeng, Jing Zhao, Hua-mao Miao, Zhi-qiang Yu, Xing-tao Zhou

**Affiliations:** 1 Department of Ophthalmology, Eye and ENT Hospital, Fudan University, Shanghai, China; 2 NHC Key Laboratory of Myopia (Fudan University), No. 83 FenYang Road, Shanghai, 200031 People’s Republic of China

**Keywords:** Monovision, SMILE, Presbyopia, Safety, Satisfaction

## Abstract

**Background:**

To investigate the safety and optical quality of small-incision lenticule extraction (SMILE) combined with monovision, and patient satisfaction with the procedure.

**Methods:**

The present study assessed a non-random case series involving 60 eyes of 30 patients (mean age 45.53 ± 3.20 years [range 41 to 52 years]) treated bilaterally using the VisuMax 500 system (Carl Zeiss Meditec, Jena, Germany) between January and July 2016. The target refraction was plano for the distance eye, and between − 0.5 and − 1.75 diopters (D) for the near eye. Visual acuity, refraction errors, ocular aberrations, and satisfaction questionnaire scores were calculated 1 year after surgery.

**Results:**

All surgeries were uneventful, with a mean safety index of 1.03 and 1.04 in dominant and nondominant eyes, respectively. Binocular uncorrected distance visual acuity of all patients was ≥20/32, while binocular uncorrected near visual acuity was ≥20/40 1 year postoperatively. Higher-order aberration (0.45 ± 0.14, 0.51 ± 0.15 μm), spherical (0.18 ± 0.15, 0.21 ± 0.14 μm) and coma aberration (0.31 ± 0.16, 0.27 ± 0.17 μm) were identical between dominant and nondominant eyes after surgery. The overall satisfaction rate was 86.7% (26/30), with large contributions from age (OR = 1.76 95% CI: 1.03–2.53; *P* = 0.036). Binocular uncorrected distance visual acuity was related to preoperative spherical diopter (*r* = − 0.500; *P* = 0.005).

**Conclusions:**

Monovision appears to be a safe and effective option for myopia patients with presbyopia who are considering the SMILE procedure. Patients with younger age were more satisfied with the procedure.

## Background

Presbyopia refers to an impairment of near vision that is common among adults > 40 years of age, resulting from declined amplitude of accommodation [1]. Currently, several surgical methods are used to correct presbyopia, including the excimer laser procedure, conductive keratoplasty, intrastromal femtosecond ring incisions, and pseudophakic multifocal intraocular lens [2]. Each procedure has advantages and disadvantages; nevertheless, surgical correction of presbyopia remains a major challenge for refractive surgeons. In recent years, refractive surgeries combined with monovision have emerged as an alternative for compensation of presbyopia, and was proven to be effective in conductive keratoplasty and laser in situ keratomileusis [3, 4]. This strategy aims to give patients both near and distance vision without glasses. It is not as invasive as multifocal intraocular changes [3], and more convenient than contact lens correction. However, reduced contrast sensitivity, reduced stereopsis, and small-angle esotropic shift associated with monovision correction were reported to be compromises after surgery [5].

With advances in refractive surgery technology, small incision lenticule extraction (SMILE) is becoming more prevalent due to its excellent safety, efficiency, and good preservation of corneal biomechanics [6, 7]. To the best of our knowledge, however, few reports have described visual outcomes of monovision induced by SMILE in myopic patients with presbyopia [8]. Accordingly, we examined monovision combined with SMILE to investigate its efficacy, safety, and patient satisfaction over a long-term follow-up period.

## Methods

The present study was a non-comparative case series, and was approved by the Ethics Committee of the Eye and ENT Hospital of Fudan University (Shanghai, China) and a written informed consent from each patient was obtained before surgery as a standard protocol preoperatively. All procedures were adhered to Declaration of Helsinki. Patients who underwent bilateral SMILE (performed by the same surgeon [ZX]) between January and July 2016, with available 1-year follow-up data, were reviewed. A total of 30 patients (10 male; mean age 45.53 ± 3.20 years [range, 41 to 52 years]) were enrolled. The cohort had a mean preoperative spherical diopter (D) of − 6.12 ± 2.39 D (− 1.5 to − 10 D), cylinder of − 0.79 ± 0.62 D (− 3.0 to 0 D), binocular uncorrected near visual acuity ranging from 20/32 to 20/20, and add 0.85 ± 0.56 D (0 to 2.25 D).

Inclusion criteria were as follows: ≥40 years of age; best corrected visual acuity ≥20/20 in either eye; spherical diopter ≤ − 10.0 D; add > 0 D; and cylinder ≤ − 3.0D. Exclusion criteria included severe eye comorbidities such as diabetic retinopathy, age-related macular degeneration, cataract causing visual impairment, or glaucoma with significant field loss, and a history of severe amblyopia or strabismus.

Regular preoperative examinations, including cycloplegic refraction, corrected visual acuity, slit-lamp examination, corneal topography (Pentacam, Oculus Optikgerate, Wetzlar, Germany), ocular aberration (WASCA wavefront analyzer, Carl Zeiss Meditec, Jena, Germany), and fundus examination were performed. The dominant eye was determined using the “hole test” [9]. Patient was asked to align a dot 4 m away through a 1″ diameter hole in a A4 sheet of paper, held at arm length. Two eyes were covered in turn, and the eye with which the dot appeared most centered was regarded as the dominant eye. The procedures above repeated at least 3 times until the result was the same for at least 2 times consecutively.

The 1-year examinations typically included manifest refraction, assessments of monocular and binocular uncorrected distance visual acuity (UDVA) (at 4 m), uncorrected neat visual acuity (UNVA) (at 33 cm) and corrected distance visual acuity (CDVA) under the same illumination. In addition, we constructed a questionnaire considering patient satisfaction including spectacle dependence for daily activities, halo, glare, visual fatigue, dry eye, and overall satisfaction [6]. Each question was graded on 4 levels: 0 indicated no discomfort whatsoever; 1 indicated discomfort occasionally occurred but did not influence life; 2 indicated discomfort, and usually influenced daily life; and 3 indicated discomfort that was too serious to tolerate. At the end of the questionnaire, patients were asked to grade overall satisfaction on a scale between 0 and 10, in which 0 indicated not satisfied at all and 10 indicated extremely satisfied.

The surgical procedure was similar to the standard SMILE treatments described by the authors in a previous study [6]. The dominant eye was corrected for distance and the nondominant eye for near, with target ranging from − 0.5 D to − 1.75 D,. Preoperatively, we used glasses to simulate target refractive status, with binocular distance visual acuity ≥20/32(the residual myopia in the nondominant eye is –X for instance). If X ≥ adding power (A), then residual myopia is set to be –A; if X ≤ A, then residual myopia is set to be –X. The overall purpose of this design was to ensure good postoperative UDVA with increasing near visual acuity as much as possible. Thus, we considered preoperative presbyopia degree only and no preventive amount of residual myopia was added into design. This principle is derived from years of surgical l experience, though individual cases will be adjusted according to the needs of life.

Statistical analysis was performed using SPSS version 22.0 (IBM Corporation, Armonk, NY, USA), and all data are presented as mean ± SD. Visual acuity data are in LogMAR units. The paired t test was performed to compare root mean square (RMS) differences in ocular aberration, and the Wilcoxon signed-rank test was performed to compare safety indexes, which were nonlinear values between the dominant and nondominant eye. For satisfaction was subjectively graded on 4 ordered levels, orderly regression analysis was used to detect factors affecting satisfaction. Factors included in this analysis are age, sex, and preoperative spherical equivalent, which are independent variables.Spearman’s test was used to determine relationships between visual acuity and other parameters; *P* < 0.05 was considered to be statistically significant.

## Results

**Fig. 1:**
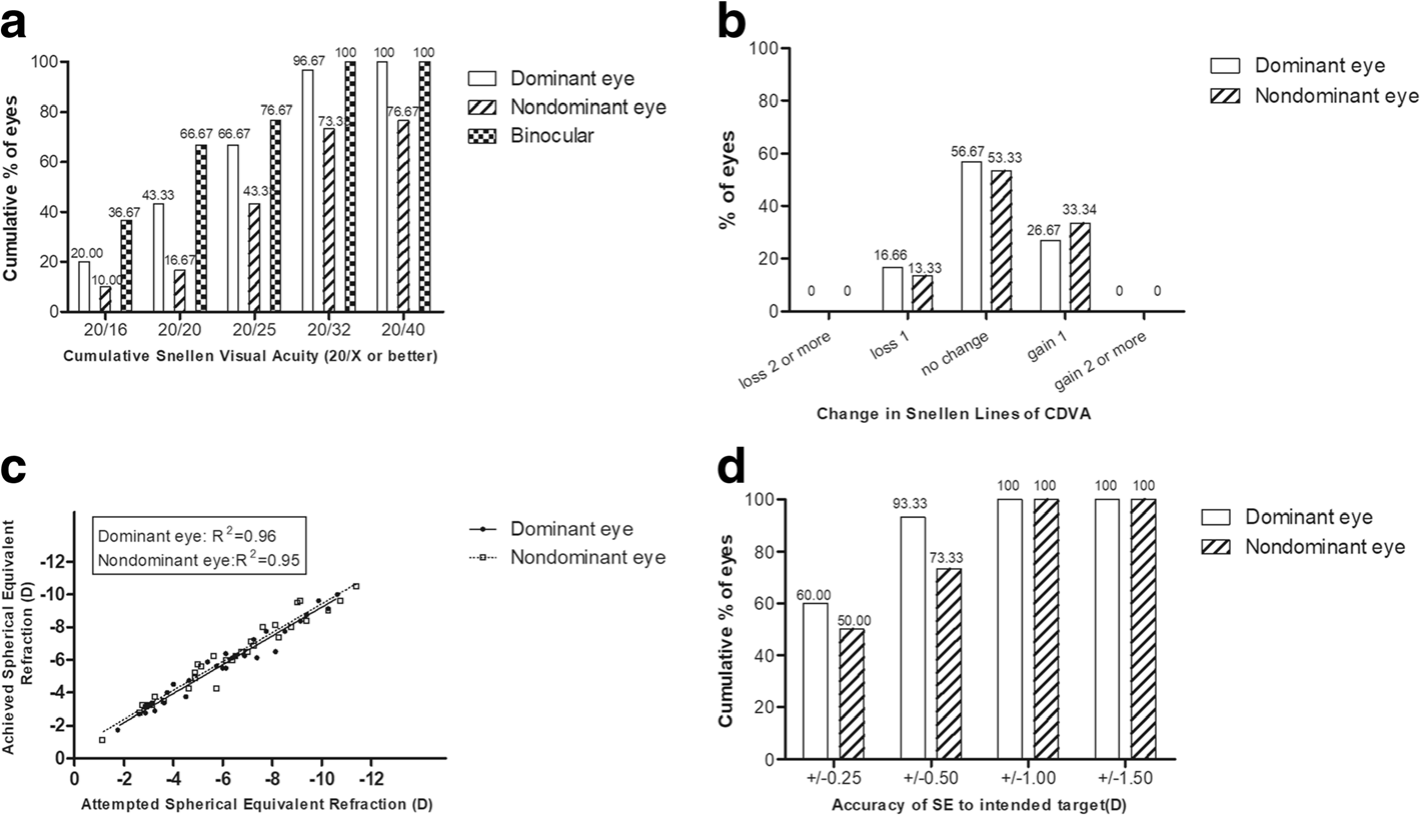
Refractive outcomes after small incision lenticule extraction combined with monovision. **a** Uncorrected Distance Visual Acuity. **b** Changes in Corrected Distance Visual Acuity. **c** Spherical Equivalent Attempted vs Achieved. **d** Spherical Equivalent Refractive vs Accuracy

All surgeries were uneventful, with no intraoperative or postoperative complications. The mean safety index was 1.03 and 1.04 (*P* > 0.05) in the dominant and nondominant eye, respectively. In the dominant eyes, the percentage of UDVA ≥20/32 were 96.7%; in the nondominant eyes, the percentage of UDVA ≥20/40 was 76.7%. Predictability and accuracy are presented in Fig. 1.

**Fig. 2:**
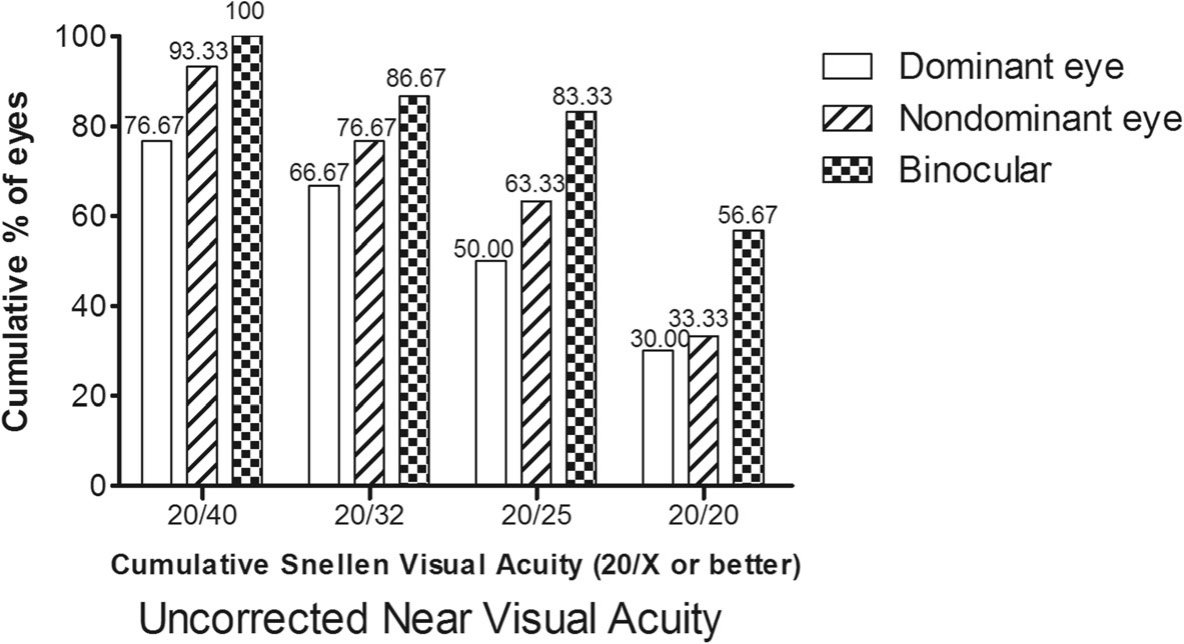
Near visual acuity after small lenticule extraction combined with monovision

As shown in Figs. 2, 93.3% of the nondominant eyes achieved UNVA ≥20/40, while 76.7% of the dominant eyes achieved UNVA ≥20/40. Binocular near visual acuity ≥20/40 was achieved in all patients.

Ocular aberrations in both eyes are summarized in Table 1. Compared with preoperative values, the RMS of total high-order aberration (HOA) and spherical aberration were not different postoperatively. Coma increased significantly after surgery (0.17 ± 0.10, 0.29 ± 0.17; *P* < 0.001) (60 eyes).

**Table 1:** Ocular aberration in dominant and nondominant eyes before and after surgery (μm)

	Preoperative (6 mm)			Postoperative (6 mm)			P^a^
	Dominant	Nondominant	P	Dominant	Nondominant	P	
HOA	0.39 ± 0.11	0.41 ± 0.33	0.896	0.45 ± 0.14	0.51 ± 0.15	0.079	0.542
SA	0.15 ± 0.07	0.12 ± 0.07	0.468	0.18 ± 0.15	0.21 ± 0.14	0.092	0.231
Coma	0.17 ± 0.10	0.13 ± 0.11	0.149	0.31 ± 0.16	0.27 ± 0.17	0.317	< 0.001

*HOA* higher-order aberration, *SA* spherical aberration

^a^Paired *t* test between preoperative and postoperative values

Results of the satisfaction survey revealed that 63.3, 6.7, and 3.3% patients experienced mild, moderate, and severe halo, respectively; 86.7% patients complained of dry eye, of which 69.2% was mild dry eye. Three patients still required reading glasses occasionally, and 6 required glasses when driving. The completely “glasses-off” rate was 76.7%. The mean satisfaction score was 8.32 ± 1.27 (range 5 to 10).

In orderly regression analysis, age, and sex constitutes a significant mode (*P* = 0.02). On that premise, age (OR = 1.76; 95% CI: 1.03–2.53; *P* = 0.036) was related to the satisfaction, while sex (*P* = 0.67) was not significant.

Spearman’s test revealed that preoperative spherical D was related to postoperative binocular UDVA (*r* = − 0.500; *P* = 0.005).

## Discussion

Monovision excimer laser correction has a considerable history in photorefractive keratectomy and laser-assisted in situ keratomileusis (LASIK), although various degrees of satisfaction have been reported in previous studies [9, 10]. With the advantages of SMILE highlighted, making use of SMILE combined with monovision has become a new treatment option for individuals with presbyopia. However, few studies have investigated the results of SMILE combined with monovision.

In our study, the mean safety index was 1.04 and 1.03 in the dominant and nondominant eye, respectively, with no statistically significant differences found between eyes. The percentage of remaining or gained BCVA was 83.3 and 86.7% in the dominant and nondominant eyes, respectively. This result is consistent with previous SMILE results [7, 11]. Levinger et al. [10] studied patients ≥40 years of age, and found that BCVA was unchanged at the 1-year follow-up. Both LAISIK and SMILE were demonstrated efficient in presbyopia treatment, though less studies about SMILE monovision are reported [8]. SMILE owes the advantage of smaller incision and less flap-related complication [12]. Accordingly, the visual quality was reported better after SMILE than LASIK, such as ocular aberration and contrast sensitivity [13]. The difference between surgeries may partly account for the various postoperative results and subjective feelings, however, direct comparison is unavailable for different criteria.

All patients in this study achieved a binocular UDVA ≥20/32, and the percentage of binocular UDVA ≥20/25 was 76.7%, which was a significant improvement from preoperative values. In terms of binocular UNVA, 100% of patients achieved UNVA ≥20/40, and the percentage of patients with UNVA ≥20/25 was 83.3%. Accordingly, SMILE combined with monovision was effective in both far and near vision. Similar results were also found in the study by Goldberg et al. [14], in which 79% of patients achieved UDVA ≥20/25, and 87.7% of patients achieved UNVA of J1 or better. However, a retreatment rate of 13.2% was reported in their study, and 5 nondominant eyes were retreated to enhance distance visual acuity. Although no patient requested retreatment in our study, distance vision loss remains a forfeit in most cases with monovision. Garcia-Gonzalez et al. [15] reported a loss in UDVA after LASIK-induced monovision. One patient in our study was dissatisfied with this surgery due to difficulty with night driving [16]. We speculate that interocular blur suppression is less effective at night and this may be a source of postoperative dissatisfaction.

Spherical aberration and HOA were unchanged after surgery, while coma increased significantly. Ocular was usually associated with postoperative glare and halo. It has been reported that SMILE-induced aberration can be restored over a long period [17]. Therefore, unchanged HOA and spherical aberration may result over a long period. Differences between the dominant and nondominant eyes were not found. A previous study reported that higher myopia errors possibly led to an increase in postoperative coma [18]. Regardless of target myopia in the nondominant eye, we found that the minor monovision would not induce unbalanced ocular aberration in both eyes, which may have contributed to postoperative satisfaction.

The satisfaction rate in this study was 86.7%, which is different from that of contact lens with monovision, which ranged from 60 to 80% in a previous study, [19] and also different from the 96% satisfaction with LASIK-induced monovision reported in the study by Goldberg et al. [14]. Further questioning of the unsatisfied patients revealed the following reasons for dissatisfaction: difficulty with night driving; visual fatigue when reading; and reduction in distance acuity. Unlike LASIK monovision, SMILE lacks the induction of spherical aberration to enhance depth of field. Though near vision is acceptable in current study, the improvement of near vison is not so obvious as the improvement of distance vision for myopic patients. Besides, the target refraction for the nondominant eye ranges from − 0.5 D to − 1.75 D, considering anisometropia tolerance for most patients. Consistent with the recommendation offered by Wright et al. [20] we are cautious about target refractions > − 2.0 D to avoid integration difficulties. Barisic et al. [3] found that − 0.5 D to − 1.25 D was suitable for presbyopic individuals < 50 years of age. Although most individuals are satisfied with this surgery, patient selection and information are critical to optimize monovision designs and warrant further study.

Although age has been considered to be unrelated to the success of monovision, [21] we found that younger individuals in the present study expressed higher satisfaction after surgery. Correspondingly, patients with early presbyopia were more satisfied. Relatively abundant accommodation reserve is helpful in acceptable UNVA. Given less surgery-induced anisometropia, patients with less severe presbyopia may have better optical quality based on a previous study [20]. Patients with higher preoperative spherical diopters tended to experience worse binocular UDVA postoperatively. Kim et al. [22] compared the efficacy of SMILE between subjects with high and mild-moderate myopia, and reported that a lower percentage of patients achieved UDVA ≥20/20 in the high-myopia group 1-year postoperatively. Coincidentally, worse predictability, efficacy and spherical aberration were found in highly myopic patients in the study by Jin et al. [23].

One limitation of the present study was the lack of a control group and, given the relatively small sample size, it was difficult to make comparisons between subgroups. Further comparison between groups stratified according to different target refraction or sex would be interesting areas of investigation. Furthermore, we only analyzed data 1-year postoperatively, and consecutive observation would be helpful to further understand the adaption period of these patients.

## Conclusions

In conclusion, SMILE combined with monovision appeared to be safe and effective in a population of presbyopic patients. Patients with younger agewere more satisfied with the procedure.

## Abbreviations

CDVA: Corrected distance visual acuity
LASIK: Laser-assisted in situ keratomileusis
SMILE: Small incision lenticule extraction
UDVA: Uncorrected distance visual acuity
UNVA: Uncorrected near visual acuity

## References

[1] Patel I, West SK (2007). Presbyopia: prevalence, impact, and interventions. Community Eye Health.

[2] Gil-Cazorla R, Shah S, Naroo SA (2016). A review of the surgical options for the correction of presbyopia. Br J Ophthalmol.

[3] Barisic A, Gabric N, Dekaris I, Romac I, Bohac M, Juric B (2010). Comparison of different presbyopia treatments: refractive lens exchange with multifocal intraocular lens implantation versus LASIK monovision. Coll Antropol.

[4] Wyzinski P (1987). Why are refractive surgeons still wearing glasses?. Ophthalmic Surg.

[5] Hayashi K, Ogawa S, Manabe S, Yoshimura K (2015). Binocular visual function of modified pseudophakic monovision. Am J Ophthalmol.

[6] Miao H, Tian M, Xu Y, Chen Y, Zhou X (2015). Visual outcomes and optical quality after femtosecond laser small incision Lenticule extraction: an 18-month prospective study. J Refract Surg.

[7] Vestergaard AH, Grauslund J, Ivarsen AR, Hjortdal JO (2014). Efficacy, safety, predictability, contrast sensitivity, and aberrations after femtosecond laser lenticule extraction. J Cataract Refract Surg.

[8] Luft N, Siedlecki J, Sekundo W, Wertheimer C, Kreutzer TC, Mayer WJ, Priglinger SG, Dirisamer M. Small incision lenticule extraction (SMILE) monovision for presbyopia correction. Eur J Ophthalmol. 2018;28(3):287–93. 10.5301/ejo.5001069 29148031

[9] Reinstein DZ, Archer TJ, Gobbe M (2011). LASIK for myopic astigmatism and presbyopia using non-linear aspheric micro-Monovision with the Carl Zeiss Meditec MEL 80 platform. J Refract Surg.

[10] Levinger E, Trivizki O, Pokroy R, Levartovsky S, Sholohov G, Levinger S (2013). Monovision surgery in myopic presbyopes: visual function and satisfaction. Optom Vis Sci.

[11] Xu Y, Yang Y (2015). Small-incision lenticule extraction for myopia: results of a 12-month prospective study. Optom Vis Sci.

[12] Arba-Mosquera S, de Ortueta D (2008). Geometrical analysis of the loss of ablation efficiency at non-normal incidence. Opt Express.

[13] Fau GS, Gupta R (2014). Comparison of visual and refractive outcomes following femtosecond laser- assisted lasik with smile in patients with myopia or myopic astigmatism. J Refrac Surg.

[14] Goldberg DB (2001). Laser in situ keratomileusis monovision. J Cataract Refract Surg.

[15] Garcia-Gonzalez M, Teus MA, Hernandez-Verdejo JL (2010). Visual outcomes of LASIK-induced monovision in myopic patients with presbyopia. Am J Ophthalmol.

[16] Chu BS, Wood JM, Collins MJ (2009). Effect of presbyopic vision corrections on perceptions of driving difficulty. Eye Contact Lens.

[17] Pedersen IB, Ivarsen A, Hjortdal J (2015). Three-year results of small incision lenticule extraction for high myopia: refractive outcomes and aberrations. J Refract Surg.

[18] de Castro LE, Sandoval HP, Bartholomew LR, Vroman DT, Solomon KD (2007). High-order aberrations and preoperative associated factors. Acta Ophthalmol Scand.

[19] Jain S, Arora I, Azar DT (1996). Success of monovision in presbyopes: review of the literature and potential applications to refractive surgery. Surv Ophthalmol.

[20] Wright KW, Guemes A, Kapadia MS, Wilson SE (1999). Binocular function and patient satisfaction after monovision induced by myopic photorefractive keratectomy. J Cataract Refract Surg.

[21] Jain S, Ou R, Azar DT (2001). Monovision outcomes in presbyopic individuals after refractive surgery. Ophthalmology.

[22] Kim JR, Kim BK, Mun SJ, Chung YT, Kim HS (2015). One-year outcomes of small-incision lenticule extraction (SMILE): mild to moderate myopia vs. high myopia. BMC Ophthalmol.

[23] Jin HY, Wan T, Wu F, Yao K (2017). Comparison of visual results and higher-order aberrations after small incision lenticule extraction (SMILE): high myopia vs. mild to moderate myopia. BMC Ophthalmol.

